# The effects of linguistic relationships among paired associates on verbal self-generation and recognition memory

**DOI:** 10.1002/brb3.98

**Published:** 2012-10-04

**Authors:** Miriam Siegel, Jane B Allendorfer, Christopher J Lindsell, Jennifer Vannest, Jerzy P Szaflarski

**Affiliations:** 1 Departments of Neurology and Environmental Health (Division of Public Health), University of Cincinnati Academic Health CenterCincinnati, Ohio; 2 Department of Neurology, University of Cincinnati Academic Health CenterCincinnati, Ohio; 3 Department of Emergency Medicine, University of Cincinnati Academic Health CenterCincinnati, Ohio; 4 Divisions of Neurology and Pediatric Neuroimaging Research Consortium, Cincinnati Children's Hospital Research Foundation, Children's Hospital Medical CenterCincinnati, Ohio; 5 Department of Neurology, Psychology, and the Center for Imaging Research, University of Cincinnati Academic Health Center; and Pediatric Neuroimaging Research Consortium, Cincinnati Children's Hospital Research Foundation, Children's Hospital Medical CenterCincinnati, Ohio

**Keywords:** Encoding, recognition memory, self-generation, word associations, word pairs

## Abstract

Previous studies have shown that self-generated information is better remembered than information that has been read passively. To further examine this subsequent memory effect, we investigated the effect of five different linguistic relationships on memory encoding. Ninety subjects were administered 60 paired associates during an encoding condition: 30 of the second words from each pair were to be read aloud and 30 were to be self-generated from clues as to the correct word. Word pairs were composed of five linguistic relationships: category, rhyme, opposite, synonym, and association. Subsequently, subjects were presented with the words that were read or generated in a forced recognition memory task. Overall, reading accuracy was higher than generation accuracy during the encoding phase (all *P* < 0.001). During the recognition phase, subjects' performance was better on the generate than on the read conditions for opposite, synonym, category, and association relationships (all *P* < 0.05), with no difference in the rhyme relationship. These results confirm previous findings that self-generated information is better remembered than read information and suggest that this advantage may be mediated by using opposite, synonym, category, and association relationships, while rhyme relationship may not extend such an advantage. These findings may have implications for future studies of memory interventions in healthy controls and subjects with cognitive impairments.

## Introduction

Previous research has shown that self-generated information is better remembered than information that is passively received (Slamecka and Graf 1978; Basso et al. 1994; Schefft et al. 2008a,b). Improved memory performance on paired associates resulting from self-generation compared with passive reading has been demonstrated in neurologically healthy adults (Slamecka and Graf 1978; Schefft and Biederman 1990; Basso et al. 1994; Vannest et al. 2012) and also in patients with traumatic brain injury (Schefft et al. 2008a), seizure disorders (Schefft et al. 2008b), Alzheimer's disease (Multhaup and Balota 1997; Barrett et al. 2000), multiple sclerosis (Chiaravalloti and Deluca 2002), Parkinson's disease (Barrett et al. 2000), schizophrenia (Vinogradov et al. 1997), and temporal or frontal lobectomy (Smith 1996). Such improvements in subsequent memory effect may be driven by multiple causes. First, an active learning process leads to improved mood state and self-esteem, and greater generalization of new knowledge (Schefft and Biederman 1990; Basso et al. 1994; Walsh et al. 1995). Individuals may be more likely to remember information because they feel self-empowered and motivated by participating in the creation of information (Olofsson and Nilsson 1992; Walsh et al. 1995). In addition, self-generated information may be better remembered because items may be made more distinctive when they are generated, leading to less memory interference (McDaniel et al. 1988). Furthermore, the generation of target words with letter deletion presents an interruption of automatic reading processes, thereby requiring an additional amount of conscious processing (McDaniel et al. 1989). Similarly, the improvements in memory performance may be driven by increased depth of semantic processing, a benefit which is not provided by structural or phonological word processing (Craik and Tulving 1975; Craik 2002; Lespinet-Najib et al. 2004). This would suggest that the improvements in memory performance due to generation would be further enhanced when semantic processing is increased, that is, when paired associates are semantically related rather than phonologically related or completely unrelated.

Several studies support the notion that semantic and phonological relationships among words are processed by separate encoding and memory mechanisms (Martin et al. 1999). For example, Martin et al. (1999) provided evidence for this concept after observing that an anomic encephalitis patient's short-term memory was characterized by an ability to normally recall digits and nonwords (i.e., phonological information) but impairment in recalling words (i.e., semantic information). Furthermore, Doré et al. (2009) demonstrated that healthy controls remembered more words that were learned in a semantic context (e.g., remembered “blueberry” when designated as a “fruit”) than those that were learned in a phonological context (e.g., remembered “bicycle” when designated as beginning with “bi”) using both free and cued recall. Additionally, Kircher et al. (2011) found that individuals were able to generate more words that fit the category of a target word than words that rhymed with a target word in a set of verbal fluency tasks; the fMRI data collected in this study showed partially overlapping, but distinctive brain networks involved in this cognitive process including left inferior frontal gyrus, middle and superior temporal gyri, and the contralateral right cerebellum in generating rhyming and categorically related words, while rhyming showed additional activation in the left inferior parietal region. Another possible explanation is that, rather than separate mechanisms, semantic and phonological relationships are processed by different allotments of cognitive resources, such as specific cognitive alignments for varying linguistic information during conversation (Menenti et al. 2012).

However, the interaction between more specific semantic and phonological memory mechanisms and the self-generation effect is not well understood. For example, Slamecka and Graf (1978) found that words generated from paired associates were better remembered than those read for all of five linguistic relationships: associations, categories, opposites, synonyms, and rhymes, but this relationship was the weakest for rhymes. Furthermore, Schefft et al. (2008b) found that epilepsy patients had significant memory improvement associated with generation specifically when encoded word pairs rhymed, in comparison to four other word-pair relationships (i.e., category, opposite, synonym, and association), illustrating that generating words with a phonological relationship may lead to better encoding in patients with memory impairment. Taken together, these studies show lack of agreement between the results of various studies and discrepancies in the processing of different linguistic relationships with the discrepancies being likely related to different approaches, different modalities tested, and relatively small sample size in some of the above-mentioned studies.

Thus, the aim of this study was to investigate the effects of five linguistic relationships between paired associates on memory of words arising from self-generation compared with passive reading in a large sample of healthy subjects. The five linguistic relationships were association, category, opposite, rhyme, and synonym. We investigated differences in the accuracy of word production from the presented word pairs for each relationship within the read and generate conditions, referred to as the encoding phase, and compared the recognition memory performance within the five different relationships for the read and generate conditions, referred to as the recognition phase. Because of differences evidenced in studies and theories on semantic versus phonological information processing (Craik and Tulving 1975; Martin et al. 1999; Schefft et al. 2008b; Doré et al. 2009; Kircher et al. 2011), we hypothesized that memory would be improved for self-generated words when compared with read words, and that this difference would be mediated by the linguistic relationship of the word pairs.

## Methods

### Subjects

This study was approved by the local Institutional Review Board and used data from 90 subjects enrolled in a larger ongoing study (NIH R01 NS04828). Subjects were male and female adults, ages 19–65, and were native English speakers with no history of neurological or psychiatric disorders. Handedness was determined using the Edinburgh Handedness Inventory, with a score of 50 or greater indicating right-handedness (Oldfield 1971). Individuals participated on a voluntary basis and were compensated for time and travel. All subjects provided written informed consent prior to study participation.

### Materials

The language paradigm used was a word-pairs task programmed and presented on a computer screen using DirectRT (Version 2008; Empirisoft, http://www.empirisoft.com). The task consisted of 60 word pairs made up of simple familiar words, each six letters or less in length (Vannest et al. 2012). Words were paired based on five different linguistic relationships, equally weighted among each of the task conditions (generate and read). The word pairs included 12 associations (e.g., “hammer–nail”), 12 synonyms (e.g., “sea–ocean”), 12 rhymes (e.g., “mist–list”), 12 opposites (e.g., “wet–dry”), and 12 category members (e.g., “sparrow–robin”) in the total list of word pairs. Six pairs within each linguistic relationship were in the read condition and six in the generate condition.

### Procedure

Behavioral data from the word-pairs task presentation were collected during and after functional magnetic resonance imaging (fMRI) performed for language localization (fMRI data presented in Vannest et al. 2012). Subjects were given task instructions and practiced the task prior to fMRI, then given task instructions again directly before performing the task during fMRI, which was the encoding phase. They were told that they would be tested on their recollection of the second word of each pair on a later test. During the encoding phase of the word-pairs task, subjects were administered word pairs on a computer screen. Thirty of the word pairs were provided entirely during the read condition (e.g., “hammer–nail”), and subjects were instructed to overtly read the second word of each pair within the 5 sec of word-pair presentation. The remaining 30 word pairs were part of the generate condition in which the first word of the pair was presented along with only the first letter of the second word followed by asterisks for the remaining letters (e.g., “spider- w**”). Subjects were instructed to generate the second word and verbalize it aloud within 5 sec of word-pair presentation. The order of read and generate trials was pseudorandom, but constant for all subjects; the word pairs assigned to each condition were presented in random order. Overt responses for each subject were recorded throughout the word-pairs task.

Within 30 min of completing the task, the subjects performed a self-paced recognition memory task (i.e., during the recognition phase) with 60 trials. The second word from every pair presented in the earlier word-pairs task (i.e., during the encoding phase) was presented simultaneously with two foil words in a forced-choice recognition task on a computer screen. Subjects were instructed to indicate which of the three words they recognized from the previous task by pressing a key corresponding to the word. The items were presented in the same order for all subjects, and the order was different from the random order of word-pair presentation they received on the earlier word-pairs task.

### Data management and analysis

Recordings of intrascanner overt responses for both the read and generate conditions were transcribed and scored to determine the proportion of correct responses during the encoding phase for each linguistic relationship and each condition. Responses for the recognition memory task were similarly scored to determine the proportion of correct responses (i.e., words correctly remembered) during the recognition phase for each linguistic relationship and each condition. The proportion of correctly remembered words came from the total list of previously presented words in the encoding phase, not simply the words read and generated aloud correctly, because subjects had the opportunity to subconsciously encode other possible responses, such as the correct word even if the incorrect word was verbally expressed aloud. In order to determine the effects of task type (word pairs versus recognition memory), response condition (read versus generate), and the interaction of the two factors for each linguistic relationship, we constructed a multivariate, full-factorial general linear model with two within subject factors (task type and response condition) for each of the five linguistic relationships (associations, categories, opposites, rhymes, and synonyms). The model was extended to test the effects of each of age, sex, and handedness on memory performance. Post hoc, paired *t*-tests were used to explore bivariate contrasts. SPSS 18.0 (SPSS Inc., Chicago, IL) was used for the statistical analysis of performance data.

## Results

**Figure 1:**
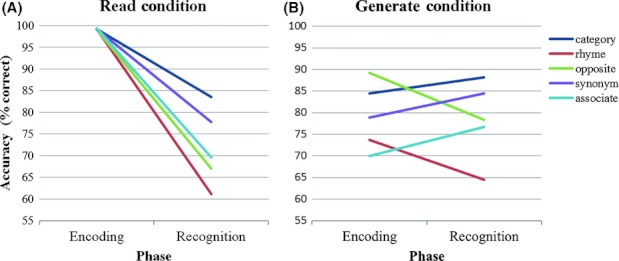
Accuracy performance trends during encoding and recognition phase for each linguistic relationship separated by read (A) and generate (B) condition. Accuracy during the encoding phase (i.e., word-pairs task) represents the proportion of words that were correctly read or self-generated. Accuracy during the recognition phase (i.e., the forced-choice recognition memory task) represents the proportion of words that were correctly identified as having been previously presented as a second word in the word pairs presented during the encoding phase.

**Figure 2:**
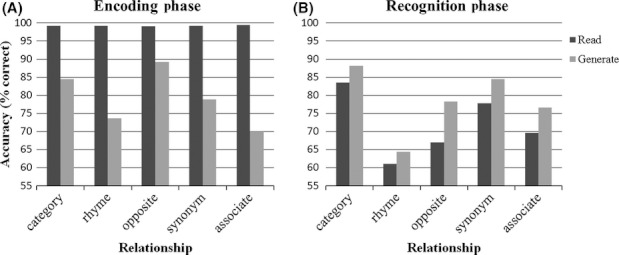
Accuracy performance for read and generate conditions by each linguistic relationship separated by encoding (A) and recognition (B) phase. Accuracy during the encoding phase (i.e., word-pairs task) represents the proportion of words that were correctly read or self-generated. Accuracy during the recognition phase (i.e., the forced-choice recognition memory task) represents the proportion of words that were correctly identified as having been previously presented as a second word in the word pairs presented during the encoding phase.

Study subjects were 39 males and 51 females. Mean age was 37.27 (SD = 13.55). There were 70 right-handed and 20 left-handed subjects. Mean performance rates for each linguistic relationship within each phase and condition are given in Table 1. Mean accuracy rates for each relationship separated by condition are shown in Figure 1 and separated by phase are in Figure 2.

**Table 1:** Mean accuracy performance for each linguistic relationship by phase and condition

Phase	Relationship	Condition	*M*	SD
Encoding	Category	Read	99.26	5.54
		Generate	84.44	16.72
	Rhyme	Read	99.26	3.45
		Generate	73.70	25.60
	Opposite	Read	99.07	3.84
		Generate	89.26	14.42
	Synonym	Read	99.26	4.26
		Generate	78.89	15.36
	Associate	Read	99.44	3.91
		Generate	70.00	21.08
Recognition	Category	Read	83.52	14.46
		Generate	88.15	14.19
	Rhyme	Read	61.11	18.86
		Generate	64.44	21.38
	Opposite	Read	67.04	18.86
		Generate	78.33	19.26
	Synonym	Read	77.78	19.03
		Generate	84.44	15.96
	Associate	Read	69.63	19.29
		Generate	76.67	19.80

The general linear model showed significant differences between the read and generate conditions, between encoding and recognition, and a significant interaction between the two (all *P* < 0.001). There were no significant effects of sex (*P* = 0.178), handedness (*P* = 0.543), or age (*P* = 0.178). In the full-factorial model including age, the main effect comparing accuracy between the encoding and recognition phases was diminished, although it remained marginally significant (*P* = 0.077). The change in the significance of effect suggests that the difference between the encoding and recognition conditions may in part be due to age.

Post hoc *t*-tests suggested that during encoding, read accuracy was significantly higher than generate accuracy for all relationships (*P* < 0.001). Conversely, during recognition, the accuracy of recalling words that were self-generated was higher than the accuracy of recalling words that were read for the synonym (*P* = 0.003), opposite (*P* < 0.001), association (*P* = 0.011), and category relationships (*P* = 0.022). Accuracy during recognition was not different between the read and generate conditions when using the rhyme relationship (*P* = 0.243). The Holm–Bonferroni approach was applied to control the familywise error rate. All comparisons remained significant with the exception of accuracy during recognition using the rhyme relationship.

## Discussion and Conclusion

Our finding that during encoding, words that were read were more accurately vocalized than words that were self-generated is expected. Simply reading words aloud is linguistically less complex than self-generating word pairs and requires less focus/attention thus decreasing the overall level of involvement in the process of encoding. Our results also confirm the previous finding that verbal material that is self-generated is more accurately remembered than material that is passively read whether it is in health or disease states (Slamecka and Graf 1978; Schefft et al. 2008b). However, in the specific case of paired associates, this self-generation effect differed depending on the linguistic relationship between the word pair. Generated words were significantly better remembered than read words in the synonym and opposite relationships, and somewhat in the category and association relationships while no recognition difference existed between read and generated words in the rhyme condition. Together, this provides support for our hypothesis that the choice of linguistic relationship has implications for memory.

As discussed above, several theories were proposed to explain the memory improvement associated with self-generation. This pattern can be attributed to the active participation that the individual is taking in achieving a task (Schefft et al. 2008b). The active learning process leads to improved mood state and self-esteem, and greater generalization of new knowledge (Schefft and Biederman 1990; Basso et al. 1994; Walsh et al. 1995). McDaniel et al. (1988) posited that self-generation improves memory because there is a heightened particularity in the items that need to be remembered. Another theory hypothesized that memory of information is enhanced when people feel self-empowered by generating that information on their own (Olofsson and Nilsson 1992; Walsh et al. 1995). In addition, generating a word with deleted letters presents an interruption of automatic reading processes, requiring an additional amount of conscious processing (McDaniel et al. 1989) leading to better recall. Furthermore, it has also been suggested that increased memory by way of self-generation is attributed to the depth of processing of the semantic information, rather than structural or phonological information (Craik and Tulving 1975; Craik 2002; Lespinet-Najib et al. 2004).

The different effects found on recognition memory for each linguistic relationship support the notion that deeper semantic processing enhances memory performance (Craik and Tulving 1975; Craik 2002; Lespinet-Najib et al. 2004); and semantic and phonological relationships among words are processed by separate memory mechanisms (Martin et al. 1999), or at least require different cognitive resources (Menenti et al. 2012). Rhyming word pairs employs phonetic knowledge to self-generate a missing word according to how the words sound, while generating other types of related word pairs, such as categories, synonyms, opposites, and associations, employs semantic knowledge of what the word actually means (Craik and Tulving 1975; Martin et al. 1999; Kircher et al. 2011). The variances in the types of word-pair relationships could possibly be explained by individuals' abilities to mentally picture the image being presented by the word's denotation (Madan et al. 2010), which would be more useful in encoding word pairs that require semantic knowledge (i.e., categories, synonyms, opposites, and associates) than phonetic knowledge (i.e., rhymes). Madan et al. (2010) found that word pairs of high “imageability,” or a greater extent to which one is able to mentally picture an object, resulted in increased memory of associations more than memory of individual items. Subjects had improved memory for associated pairs than for separate items when words were characterized by high imageability. The current study employed related word pairs, and therefore, memory of these pairs could have benefited from the ability to image the association. It is plausible, therefore, that differences in memory performance between the linguistic relationships can be related to the imagery of those word pairs presented within them.

Memory performance was not statistically different between read and generate conditions when a rhyming linguistic relationship was used. This illustrates that verbal self-generation may not universally improve memory compared with passively reading words, and that linguistic relationship plays a role in effective memory formation. That rhyming was the linguistic relationship demonstrating least differences between the read and generate conditions is inconsistent with previous findings in which epilepsy patients demonstrated improved memory performance for generated words of a rhyming relationship when compared with categories, synonyms, opposites, and association (Schefft et al. 2008b). This could be explained by difference in populations. As the current study employed neurologically intact subjects, the study by [Bibr b21][Bibr b22]) enrolled epilepsy patients who usually present with increased memory complaints when compared with healthy population (Kent et al. 2006; Black et al. 2010), and thus, these subjects may benefit more from memory improvement exercise. Another explanation may be that different linguistic relationships may be more effective for recognition memory in different study populations (Eliassen et al. 2008). Another study found that healthy individuals better remembered words they self-generated than passively read from all five linguistic relationships (Slamecka and Graf 1978), whereas the current study found these results in all of the relationships except rhyme. However, the former study enrolled a small number of subjects who were informed on the linguistic relationship being administered for each word pair and presented word pairs blocked by that relationship, whereas the current study presented word pairs in random order without informing which relationship was being employed. Presenting word pairs according to linguistic relationship could have cued subjects to encode and remember words differently, which could account for the recognition differences. These procedural discrepancies may account for the relatively minor differences between these studies although it appears that rhyming words may be processed differently than semantically related words.

Of the relationships in which self-generated words were better remembered than passively read words, we observed qualitatively that words from the category member relationship resulted in the best memory performance, followed by synonym, opposite, and associate in the generate condition (Fig. 1B). Words from the rhyme relationship displayed the worst memory performance. This is consistent with the results of the study by Doré et al. (2009), which found that healthy controls remembered more words that were encoded in a semantic context than those that were encoded in a phonological context using both free and cued recall. Additionally, Kircher et al. (2011) found that subjects were more successful in generating words in the same category of a target word than words that rhymed with a target word. Furthermore, Slamecka and Graf (1978) also found that rhyming word pairs showed the overall lowest recognition performance. Of interest, memory performance was similarly ordered for both the generate and read conditions; words of the category relationship resulted in the highest memory performance, followed by synonym, associate or opposite, and rhyme (Fig. 2B). This may suggest that the techniques employed in recognizing words are similar for read and self-generated words. However, our study was not designed to place memory performance in order by linguistic relationship, so these observations should be interpreted with caution.

Overall, our study showed that category members, synonyms, opposites, and associates were relationships that promoted the highest memory performance. Within each of these relationships, self-generated words were better remembered than passively read words. Self-generation of words from semantic word-pair relationships, such as categories, synonyms, opposites, and associates, may improve memory performance. This could have applications in many aspects of memory enhancement. For example, the technique of self-generating information, and applying specific linguistic relationships, could benefit study methods in vocabulary and language learning. These results could also contribute to the development of language and memory therapies for patients with neurological disorders if the results were replicated in patient populations such as patients with traumatic brain injury (Schefft et al. 2008a), seizure disorders (Schefft et al. 2008b), Alzheimer's disease (Multhaup and Balota 1997; Barrett et al. 2000), multiple sclerosis (Chiaravalloti and Deluca 2002), Parkinson's disease (Barrett et al. 2000), schizophrenia (Vinogradov et al. 1997), and lobectomy (Smith 1996). We note that the benefits to memory found in the present study are limited to cued recognition memory. Future studies should consider different memory tasks, such as free recall, to investigate the consistency of effects of linguistic relationships of paired associates on memory performance and assessment of the short- versus long-term effects of paired encoding on memory performance. Finally, neuroimaging results could be analyzed to examine if differences in neural circuits exist between the five linguistic relationships as seen in the behavioral results.

In conclusion, we show that self-generated information is better remembered than passively read information using a cued-recall task; and memory performance is impacted by the linguistic relationship employed, with a rhyming relationship differing in performance to semantic relationships. These findings can be used to guide memory enhancement and, if extended to neurologically impaired persons, perhaps treatment.
